# Recent Research on Structural Design, Performance Optimization, and Applications of Piezoelectric Pumps

**DOI:** 10.3390/mi16040474

**Published:** 2025-04-16

**Authors:** Qiufeng Yan, Zhiling Liu, Le Wang, Wanting Sun, Mengyao Jiang

**Affiliations:** 1 School of Electrical Engineering and Automation, Nantong University, Nantong 226019, China; yanqf@nuaa.edu.cn; 2 School Key Laboratory of Mechanics and Control for Aerospace Structures, Nanjing University of Aeronautics and Astronautics, Nanjing 210016, China; 3 Graduate School of Education, Joongbu University, Goyang 10279, Republic of Korea; 4 Huzhou Institute of Zhejiang University, Huzhou 313000, China; 2023019@huvtc.edu.cn; 5 School of Engineering, Lancaster University, Lancaster LA1 4YW, UK; sunwt_hit@126.com

**Keywords:** PP, structural classification, optimization, application

## Abstract

With the advantages of simple structure, low power consumption, no electromagnetic interference, and fast response, piezoelectric pumps (PPs) have been widely used in the fields of chip cooling, biomedical applications, chemical applications, and fuel supply applications. In recent decades, scholars have proposed various PPs, and this article reviews the recent research results. In this review, according to the “valve” structure, PPs are divided into valve-less piezoelectric pumps (VLPPs), valve-based piezoelectric pumps (VBPPs), and piezoelectric pumps with valve and valve-less state transitions (PPVVSTs). Firstly, the design methods of typical structures were discussed, and comparisons were made in terms of driving frequency, driving voltage, output pressure, flow rate, structure materials, and pump size. The advantages and disadvantages of VLPPs, VBPPs, and PPVVSTs were analyzed. Then, we compared the driving parameters, output performance, structure materials, and pump size of single-chamber piezoelectric pumps (SCPs) and multi-chamber piezoelectric pumps (MCPs) and analyzed the advantages and disadvantages of SCPs and MCPs. Optimization methods proposed in recent years have been summarized to address the issues of the cavitation phenomenon, the liquid back-flow problem, and low output performance in PPs. Subsequently, the application research of PPs and the distribution of academic achievements were discussed. Finally, this review was summarized, and future research hot spots for PPs were proposed. The main contribution of this review is to provide piezoelectric pump (PP) researchers with a certain understanding of the structural design, optimization methods, practical applications, and research distribution of PPs, which can provide theoretical guidance for future research.

## 1. Introduction

**Figure 1:**
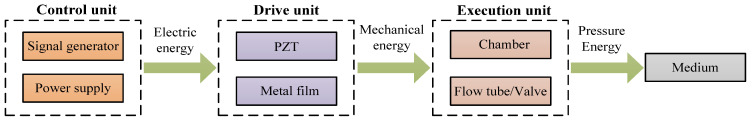
Schematic diagram of energy conversion of PP.

The piezoelectric pump (PP) [1,2,3,4,5] is a device that utilizes the inverse piezoelectric effect of piezoelectric materials to convert electrical energy into mechanical energy for fluid transportation. It mainly includes a control unit, a driving unit, and an execution unit. The control unit consists of a signal generator and a power supply. The driving unit is composed of piezoelectric ceramics and metal films, which can convert electrical energy into mechanical energy and serve as the power source for PPs. The execution unit is composed of a pump chamber and a flow tube or valve, mainly converting mechanical energy into kinetic energy of the fluid to achieve a unidirectional flow of the fluid. Figure 1 shows the schematic diagram of the energy conversion of a PP.

In 1978, Narasaki [6] was the first to propose a PP—a pump which achieved the unidirectional flow of liquids by combining piezoelectric materials with special structures. In comparison with traditional pumps, PPs have the advantages of simple structure, low energy consumption, no electromagnetic interference, and fast response [7], etc. Because of these numerous advantages, many scholars have conducted research on PPs. Currently, PPs have been widely used in chip cooling [8,9,10], biomedical applications [11,12,13], chemical analysis [14,15,16], and fuel supply applications [17,18,19].

**Figure 2:**
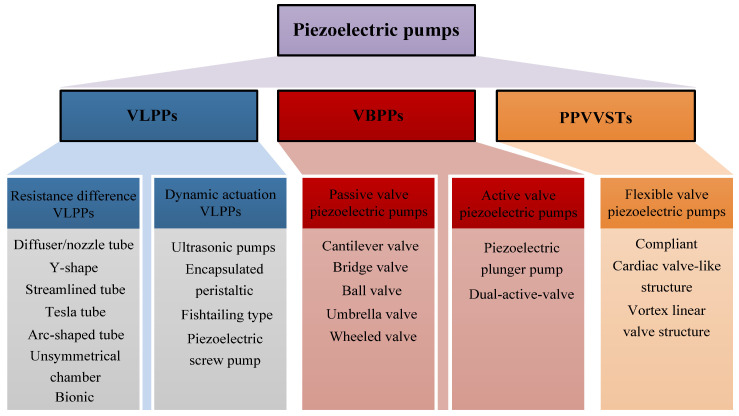
Structural classifications of PPs.

Based on the valve body structure, PPs can be divided into VLPPs [20] and VBPPs [21]. VLPPs have no movable valve body, which means that they can adapt to higher driving frequencies and more complex working conditions. However, there is a liquid back-flow problem in VLPPs [22], resulting in low a pumping efficiency and output flow rate. VBPPs can achieve a high output pressure and flow rate, but they are prone to fatigue and wear. When the frequency is too high, the lagging of valves may even occur [23]. To address the structural deficiency problems of VLPPs and VBPPs, Fu et al. [24] proposed a semi-flexible valve piezoelectric pump that can achieve the transition between the “valve-less” and “valve-based” working states of PPs under different driving conditions according to the application scenario requirements, thereby expanding the application fields of PPs. Figure 2 shows the structural classifications of PPs, which can be classified as VLPPs, VBPPs, and PPVVSTs. VLPPs include resistance difference VLPPs and dynamic actuation VLPPs. VBPPs include passive valve PPs and active valve PPs.

This paper summarizes the types of PPs, reviews the typical structural characteristics of PPs, summarizes the optimization methods of PPs, discusses the potential applications of PPs, and analyzes the distribution of research results on PPs. According to the current status of the research, the future development prospects of PPs have been proposed, which lay a theoretical foundation for the further application of PPs.

## 2. Classification of PPs

### 2.1. VLPPs

VLPPs can achieve one-way fluid flow through asymmetric channels or ultrasonic vibration. According to the working principles of VLPPs, they can be classified as resistance difference VLPPs and dynamic actuation VLPPs.

#### 2.1.1. Resistance Difference VLPPs

**Figure 3:**
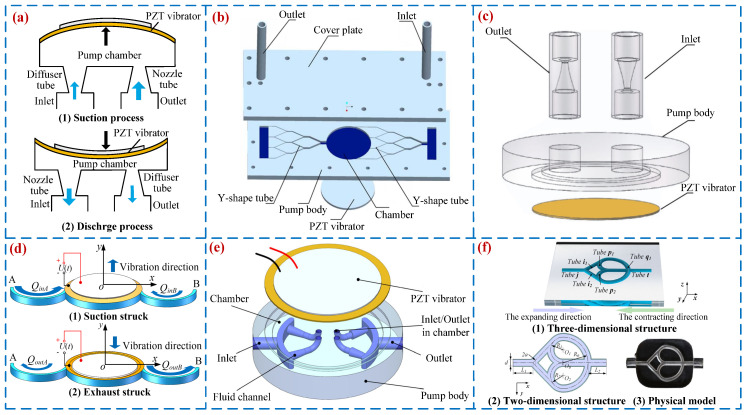
Resistance difference VLPPs: (**a**) a diffuser/nozzle tube by Stemme et al. [25], (**b**) a multistage Y-shaped tube by Huang et al. [31], (**c**) a streamlined tube by Bian et al. [32], (**d**) an arc-shaped tube by Yan et al. [36], (**e**) a four-coned tube by Zhang et al. [39], and (**f**) a double-looped tube by Chen et al. [40].

In 1993, Stemme et al. [25] first presented a resistance difference VLPP with diffuser/nozzle tubes (see Figure 3a). Olsson et al. [26] proposed a parallel structure consisting of two pump chambers and a diffuser/nozzle tube fixed on the same plane and optimized the structure of a VLPP with diffuser/nozzle tubes to improve efficiency. Verma et al. [27] studied the effects of excitation signals and structural parameters on the performance of PPs. By changing the excitation signal and structural parameters, fluid can achieve forward, reverse, and two-way flow. In addition, Tseng et al. [28], Aggarwal et al. [29], and others have conducted research on VLPPs with diffuser/nozzle tubes. To meet the high survival rate of transporting live cells and long-chain polymers, Zhang et al. [30] proposed a VLPP with Y-shaped tubes, which greatly reduces the generation of eddy currents compared with a VLPP with diffuser/nozzle tubes. Huang et al. conducted research on VLPPs with diffuser/nozzle tubes and proposed a VLPP with multistage Y-shaped tree-like bifurcate tubes (see Figure 3b) [31]. In 2020, Bian et al. [32] proposed a VLPP with streamlined flow tubes based on the characteristics of streamlined curves (see Figure 3c) and further optimized the structure of the streamlined flow tube [33]. In addition to the above research, scholars have also studied the following types of resistance difference VLPPs: a VLPP with Tesla tubes [34], a VLPP with cross-shaped tubes [35], a VLPP with arc-shaped tubes (see Figure 3d) [36], a VLPP with rotatable unsymmetrical slopes [37,38], a VLPP with four-coned tubes (see Figure 3e) [39], and a VLPP with double-looped tubes (see Figure 3f) [40]. Figure 3 illustrates the structures and working principles of resistance difference VLPPs.

#### 2.1.2. Dynamic Actuation VLPPs

**Figure 4:**
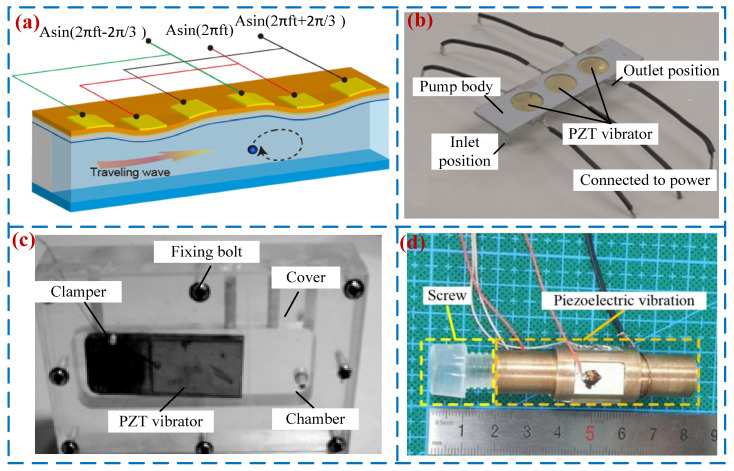
Dynamic actuation VLPPs: (**a**) an ultrasonic by Ogawa et al. [42], (**b**) a peristaltic by Zhang et al. [43], (**c**) a bionic by Huang et al. [44], and (**d**) a screw by Yin et al. [45].

In 2000, Rife et al. [41] proposed a miniature valve-less ultrasonic pump that generates vibration energy through piezoelectric driving elements and applies instantaneous pressure to the fluid to achieve the function of the pump. With the development of new materials and micro/nano fabrication technologies, piezoelectric ultrasonic pumps are becoming increasingly mature. Ogawa et al. [42] proposed a piezoelectric ultrasonic pump (see Figure 4a) that utilizes the piezoelectric thin film to generate traveling waves in micro-channels for fluid transport; a peak flow rate of 0.172 mL/min was achieved when the voltage was 10 V_p-p_ at 600 kHz. Zhang et al. [43] designed an encapsulated peristaltic piezoelectric micro-pump using a multi-layer ceramic preparation method (see Figure 4b), and a peak flow rate of 0.45 mL/min and pressure of 1.4 kPa were obtained at the voltage and frequency of 100 V_p-p_ and 100 Hz. Huang et al. [44] presented a fishtailing type of VLPP with a rectangular vibrator (see Figure 4c), and a flow rate of 6.4 mL/min was achieved under the voltage of 100 V_p-p_ at 460 Hz. Yin et al. [45] designed a novel piezoelectric screw pump (see Figure 4d) that uses piezoelectric ceramics (PZT) to drive the threaded tube to exhibit rotational motion, pushing the liquid to be pumped in the direction of the thread. Therefore, the output flow rate and back pressure of the VLPPs are improved. A peak flow rate of 0.75 mL/min was achieved when the voltage was 800 V_p-p_ at 13.8 kHz. Figure 4 shows the structures and working principles of dynamic actuation VLPPs. Table 1 presents the properties for VLPPs, including the first author and year, type of valve, structure material, volume, voltage, frequency, pressure, and flow rate.

### 2.2. VBPPs

#### 2.2.1. Passive Valve PPs

**Figure 5:**
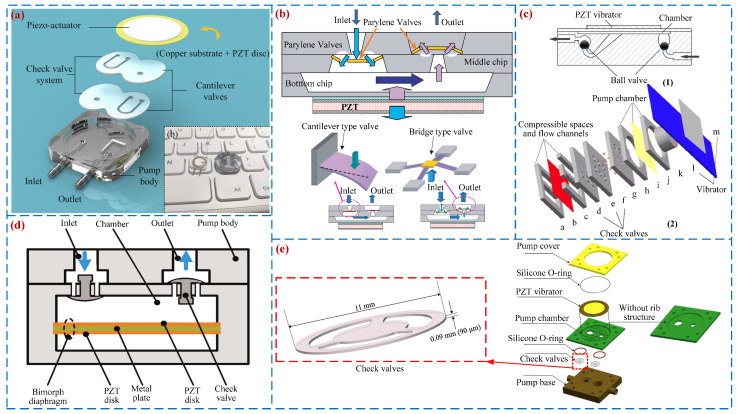
Passive valve PPs: (**a**) a cantilever valve by Yang et al. [52], (**b**) a bridge valve by Feng et al. [53], (**c**) a ball valve by Carrozza et al. [54] and Pan et al. [55], (**d**) an umbrella valve by Zhang et al. [56], and (**e**) a wheeled valve by Ma et al. [57].

Passive valve PPs open and close valves through periodic pressure inside the pump chamber. They have the advantages of compact structure, low power consumption, and easy processing and have attracted widespread attention from scholars [21]. In 1988, Van et al. [46] first proposed a passive valve PP based on silicon-based processing technology. The valve types of passive valve PPs include: Cantilever valve [47], Bridge valve [48], Ball valve [49], Umbrella valve [50], Wheeled valve [51], etc. When the cantilever valve is used as a one-way valve for PPs, it has the advantages of simple structure and easy assembly. Young et al. [52] designed a high-performance, small volume, and low-cost PP using the cantilever valve (see Figure 5a). The volume of the pump is only 11 × 11 × 1.5 mm, but its maximum output pressure and flow rate can reach 52 kPa and 4.5 mL/min. Feng et al. [53] proposed a bridge valve PP (see Figure 5b), and a peak flow rate of 0.7 mL/min and pressure of 4 kPa were achieved when the voltage was 100 V_p-p_ at 6 kHz. In 1995, Carrozza et al. [54] used stereolithography technology to fabricate a ball valve piezoelectric pump (see Figure 5c) with a diameter of 1.2 mm and a flow rate of 46 μL/min. Considering the high reliability and low-cost advantages of ball valves, Pan et al. [49], Wu [55], and others have conducted research on ball valve PPs. In 2015, Zhang et al. [56] developed an umbrella valve PP (see Figure 5d) that simultaneously achieves fluid transfer and self-induction functions; the flow rate of 28.71 mL/min was obtained under the voltage of 300 V_p-p_ and frequency of 21 Hz. In the same year, Ma et al. [57] designed a wheeled one-way valve PP (see Figure 5e), and a peak flow rate of 196 mL/min was achieved when the voltage was 70 V_p-p_ at 25 kHz. Figure 5 shows the structures and working principles of passive valve PPs.

#### 2.2.2. Active Valve PPs

**Figure 6:**
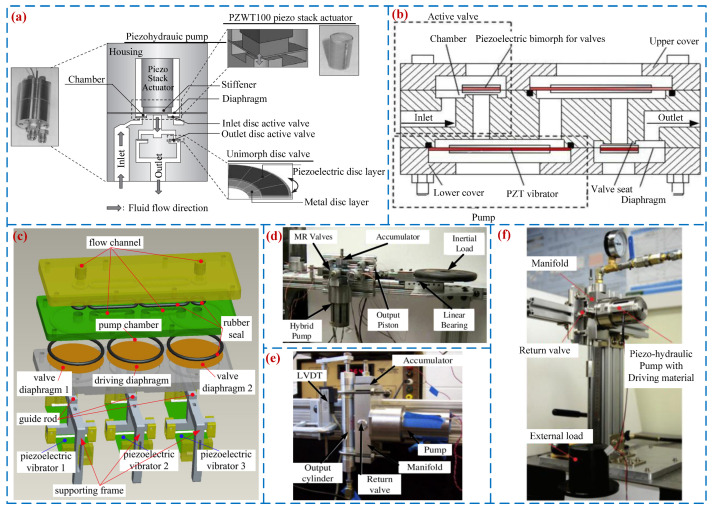
Active valve PPs: (**a**) a PP by Lee et al. [58], (**b**) a PP by Cheng et al. [59], (**c**) a PP by Sun et al. [60], (**d**) an EHA by Wereley et al. [63], (**e**) an EHA by Wereley et al. [64], and (**f**) an EHA by Xuan et al. [66].

In order to improve the lagging of valves of passive valve PPs, scholars have proposed active valve PPs, which actively control the opening and closing of the valve body inside the pump through external devices, achieving synchronous operation of the valve body and PZT vibrator. Lee et al. [58] realized synchronous motion between the PZT vibrator and the valve plate by developing an active valve PP (see Figure 6a) that improved the lagging of valves and achieved a flow rate of 204 mL/min under 1 kHz. Cheng et al. [59] designed a PP with an active valve actuated by a rectangular piezoelectric vibrator (see Figure 6b), and the peak flow rate was 140 mL/min when the voltage was 75 V_p-p_ at 70 Hz. Sun et al. [60] proposed a two-way active valve piezoelectric pump for precision fluid transport (see Figure 6c). The piezoelectric stack pump is one of the most important branches of the active valve PPs. It has the advantages of high operating frequency and large output pressure. It can convert high-frequency, pulsed mechanical motion into low-frequency, semi-continuous fluid motion. Therefore, many scholars have carried out research on the piezoelectric stack pumps. In 2000, Lynch et al. [61] developed a piezoelectric–hydraulic hybrid actuator that achieved a flow rate of 5.16 mL/min and a blocked pressure of 1.6 MPa when driven at 1000 V with a frequency of 10 Hz. They also established a state model for the piezoelectric stack pump [62], conducting partial simulation and experimental studies on the hydraulic system. In 2008, Wereley et al. [63] designed a magnetorheological fluid valve based on magnetorheological fluid and applied it to a Terfenol-D-driven actuator (Figure 6d). The maximum output flow rate was 0.754 L/min. Subsequently, Wereley et al. [64] employed Terfenol-D materials with two different lengths to drive a hybrid electro–hydrostatic actuator (EHA) (Figure 6e), attaining a peak flow rate of 1.488 L/min. Wereley et al. [65] designed a compact hybrid electro–hydrostatic actuation system using electrostrictive material (PMN-32%PT) and studied the variation law of its output performance under the driving signal. Experiments showed that the no-load peak flow rate of this actuator was 2.55 L/min. Xuan et al. [66] developed a piezoelectric–hydraulic hybrid EHA driven by a piezoelectric stack (Figure 6f), designed to meet the requirements of high-performance and compact flight systems. This system achieved a maximum output flow rate and maximum power of 1.023 L/min and 8.74 W, respectively. Wang et al. [67] proposed a novel active valve PP, which mainly consists of a PP, a hydraulic cylinder, and a valve. The pump can be used in high-pressure environments, and the flow rate is 3000 mL/min at 150 Hz. Figure 6 exhibits the structures and working principles of active valve PPs. Table 2 gives the properties for VBPPs, including the first author and year, type of valve, structure material, volume, voltage, frequency, pressure, and flow rate.

### 2.3. PPVVSTs

**Figure 7:**
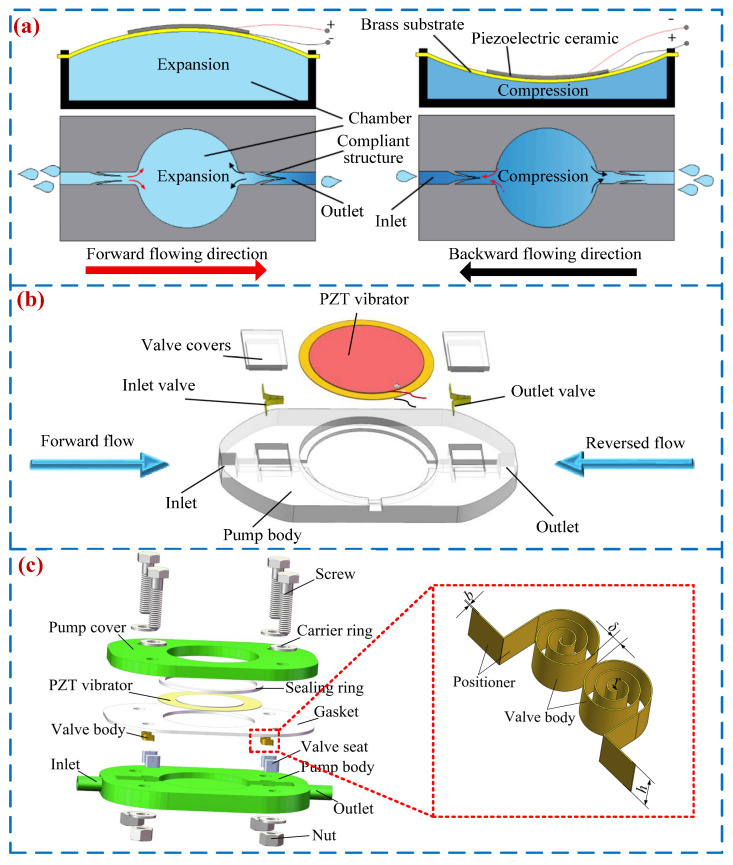
PP with valve and valve-less state transition: (**a**) a PPVVST by Bao et al. [68] and Fu et al. [24], (**b**) a PPVVST by Huang et al. [69], and (**c**) a PPVVST by Yan et al. [71].

In 2019, Bao et al. [68] and Fu et al. [24] designed a flexible valve PP (see Figure 7a). The pump achieves one-way transmission of liquid by incorporating flexible valve bodies on both sides of the flow channel. When the voltage is below a certain value, the working state of the PP is “valve-less”, and when the voltage is above a certain value, the working state of the PP is “valve-based”. The output pressure and flow rate of the PP have two peak values. Huang et al. [69] developed a novel flexible valve PP (see Figure 7b), and a peak flow rate of 119.61 mL/min and pressure of 6.16 kPa were achieved when the voltage was 100 V_p-p_ at 25 Hz. Zhou et al. [70] designed a PP with a cardiac valve-like structure based on the cardiac valve structure, and a peak output pressure and flow rate were 199 kPa and 44.5 mL/min. To enhance the output performance of the flexible valve PPs, Yan et al. [71] designed a PP with a vortex linear valve structure, which increased the average flow rate by 107%; a peak flow rate of 44.3 mL/min was obtained under the voltage of 180 V_p-p_ and frequency of 12 Hz. Figure 7 shows the structures and working principles of PPVVSTs. Table 3 exhibits the properties for PPVVSTs, including the first author and year, type of valve, structure material, volume, voltage, frequency, pressure, and flow rate.

### 2.4. Brief Analyses of VLPPs, VBPPs, and PPVVSTs

**Figure 8:**
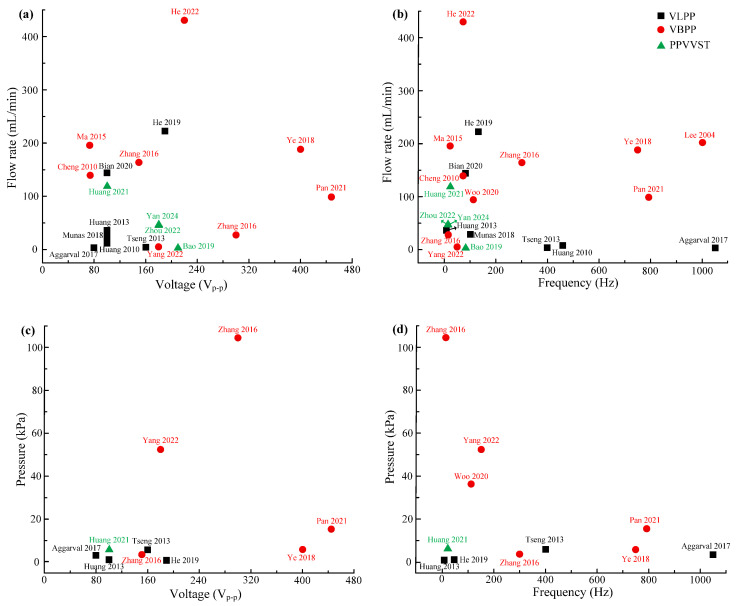
Maximum flow rates and pressures for VLPPs, VBPPs, and PPVVSTs: (**a**,**b**) flow rates versus voltages and frequencies and (**c**,**d**) pressures versus voltages and frequencies [28,29,31,32,35,38,44,47,48,49,50,51,52,56,57,58,59,68,69,70,71].

Figure 8 shows the peak output pressures and flow rates of VLPPs, VBPPs, and PPVVSTs. VLPPs have low flow rates and output pressures—the output flow rates of the VLPPs are less than 150 mL/min, and the output pressures are less than 10 kPa. VLPPs are suitable for fields such as biomedical applications, chemical analysis, etc. However, VLPPs are prone to fluid back-flow problems and have a low working efficiency. VBPPs have high flow rates and output pressures—the output flow rates of the VBPPs are over more than 100 mL/min, and the output pressures are over more than 20 kPa. VBPPs are suitable for fields such as fuel supply, chip cooling, hydraulic system braking, etc. However, under high-frequency driving, the valve body is prone to damage and wear, seriously affecting the service life of PPs. The valve structure of VBPPs can damage the structure of living cells, limiting the further application of PPs. In addition, VBPPs are affected by “lagging of valve [23]”. The emergence of PPVVSTs can effectively solve the “fluid back-flow” problem of VLPPs and the “lagging of valve” problem of VBPPs. By changing the driving parameters, the transition between the “valve-less” and “valve-based” working states of PPs can be achieved, making PPs applicable to more application fields.

## 3. Chamber Configuration of PPs

The PZT vibrator and pump chamber are the key components of piezoelectric pumps, and their combination can provide a power source for the PPs [7]. Based on the number of pump chambers, PPs can be divided into SCPs [72] and MCPs [73]. According to the arrangement of pump chambers, MCPs can be divided into multi-chamber series-type PPs [74], multi-chamber parallel-type PPs [75], and multi-chamber hybrid-type PPs [76].

### 3.1. SCPs

**Figure 9:**
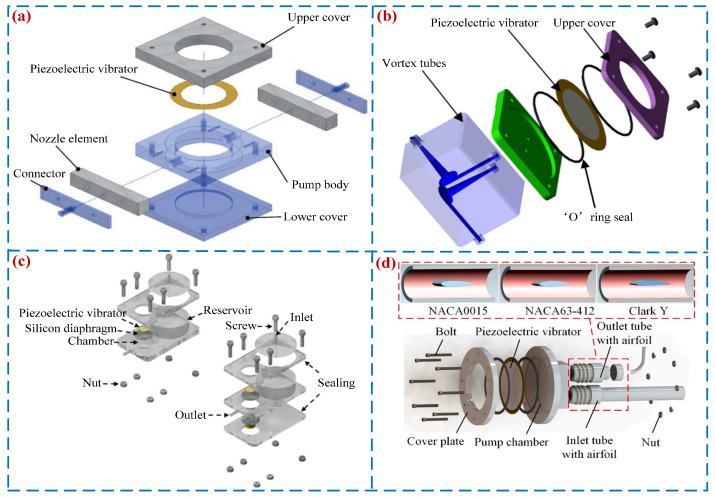
Single-chamber PPs: (**a**) an SCP by Choi et al. [79], (**b**) an SCP by Huang et al. [77], (**c**) an SCP by Dereshgi et al. [80], and (**d**) an SCP by Huang et al. [81].

SCPs have attracted the attention of many scholars due to their simple structure and easy processing and control [35,77]. Since Spencer et al. [78] first proposed PPs in 1978, SCPs have experienced rapid development. Researchers have developed various forms of SCPs, in which the PPs with the diffuser/nozzle tubes [25] have become the most common. Choi et al. [79] designed a low-cost VLPP (see Figure 9a), and a peak output pressure and pump flow rate were 0.93 kPa and 23 mL/min. Huang et al. [77] designed a VLPP with vortex tubes (see Figure 9b), and a peak flow rate of 27.5 mL/min and pressure of 0.17 kPa were achieved when the voltage was 100.0 V_p-p_. Dereshgi et al. [80] developed single-diaphragm and double-diaphragm micro-pumps (see Figure 9c) that can pump liquids through a pumping chamber and a fixed reservoir; the peak flow rate was 35.4 mL/min. Huang et al. [81] proposed a high flow SCP based on the characteristics of the wing (see Figure 9d), and a peak flow rate of 235.56 mL/min and pressure of 0.843 kPa were obtained at the voltage and frequency of 100 V_p-p_ and 47 Hz. Figure 9 shows the structures and working principles of SCPs.

### 3.2. MCPs

**Figure 10:**
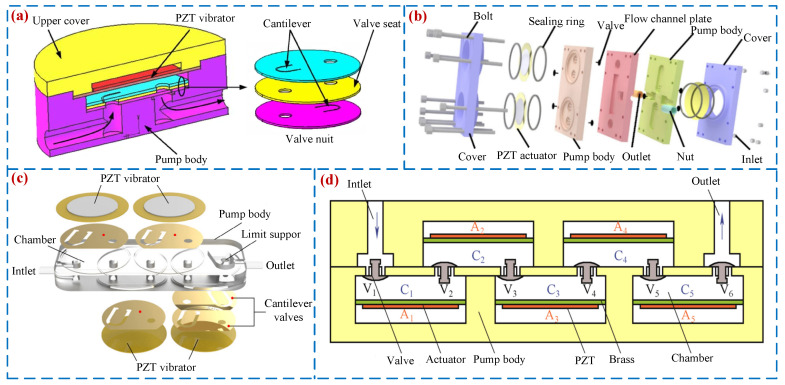
MCPs: (**a**) an MCP by Kan et al. [83], (**b**) an MCP by Hu et al. [84], (**c**) an MCHP by Liu et al. [85], and (**d**) an MCPP by Zhang et al. [74].

To enhance the output performance of the PPs, Olsson et al. [82] optimized the VLPP with diffuser/nozzle tubes and proposed a dual-chamber parallel PP, where the peak output pressure and flow rate reached 16.5 kPa and 16 mL/min. Considering the advantages of MCPs in improving performance, more and more scholars are paying attention to the research of MCPs. Kan et al. [83] proposed a cantilever valve dual-chamber PP (see Figure 10a), and a peak flow rate of 3.0 mL/min and pressure of 9.0 kPa were achieved when the voltage was 50 V_p-p_ at 200 Hz. Hu et al. [84] designed a cascade-type three-chamber PP (see Figure 10b), where the peak output pressure and flow rate reached 19.38 kPa and 466 mL/min. A four-chamber hybrid-type PP (see Figure 10c) was designed by Liu et al. [85], which has significantly advanced output performance compared with dual-chamber and three-chamber PPs. The maximum output pressure and flow rate reached 140 kPa and 110 mL/min. Zhang et al. [74] developed a six-chamber series-type PP (see Figure 10d), the peak flow rate of 469.2 mL/min was achieved when the voltage was 150 V_p-p_ at 400 Hz. Lu et al. [86] designed a ten-chamber PP, where the peak output pressure and flow rate reached 109.9 kPa and 251.1 mL/min. There are no reports on further chamber PPs in subsequent research. Figure 10 shows the structures and working principles of MCPs.

### 3.3. Brief Analyses of SCPs and MCPs

**Figure 11:**
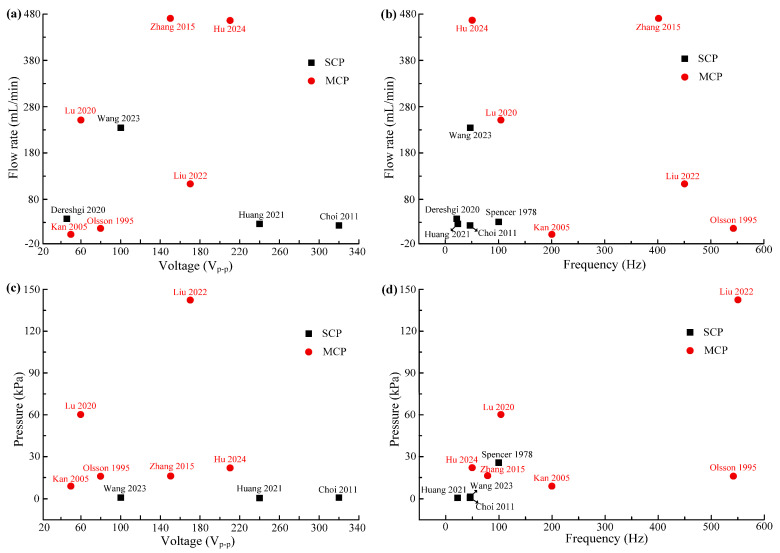
Maximum flow rates and pressures for single-chamber PPs and multi-chamber PPs: (**a**,**b**) flow rates versus voltages and frequencies and (**c**,**d**) pressures versus voltages and frequencies [74,77,78,79,80,81,82,83,84,85,86].

Table 4 exhibits the properties for SCPs and MCPs, and Figure 11 shows the peak output pressures and flow rates of SCPs and MCPs. The SCP has only one pump chamber, with simple structure but limited output performance. The output pressures of the SCPs were less than 30 kPa, and the flow rates were less than 100 mL/min. Scholars usually improve the output performance of PPs by increasing the driving voltage [2,87,88], but excessive driving voltage can cause damage to the PZT vibrator [89]. Researchers have conducted extensive research on improving the output performance of PPs [85,90] and have found that increasing the number of pump chambers can enhance the output performance of PPs. Lu et al. [86] found that the series and the parallel connection of pump chambers can affect the output performance of PPs. PPs can obtain the maximum flow rate when the pump chambers are connected in a series, and the maximum output pressure can be obtained when the pump chambers are connected in a mix. In addition, according to Table 4 and Figure 11, MCPs have a significant advantage in output performance compared with SCPs. The output flow rates of the MCPs were over more than 450 mL/min, and the output pressures were over more than 140 kPa. However, with the disadvantages of such a complex structure, high cost, and difficulty in processing, MCPs have not been further promoted and miniaturized.

In summary, in the research of piezoelectric pumps, it is necessary to find a balance point between the number of pump chambers, the overall structure of the PPs, and the output performance of the PPs. While the output performance meets the requirements, the complexity of the structure and the number of pump chambers should be minimized as much as possible.

## 4. Optimization of PPs

In order to improve the output performance of the PPs, researchers have presented many optimization methods, including cavitation inhibition, back-flow suppression, and optimization of the PZT vibrator.

### 4.1. Cavitation Inhibition Methods

Cavitation [91] is a fluid mechanics phenomenon widely present in fluid machinery and other industrial equipment, especially in pumps. A PP is a high-frequency vibration mechanical system, and the cavitation phenomenon can change the flow characteristics of the fluid inside the pump, thereby affecting the output performance of the PP [92,93]. Opitz et al. [94] found that cavitation in piezoelectric reciprocating pumps begins in the initial stage of the suction phase. In 2006, Zhang et al. [95] designed an anti-cavitation method based on a three-dimensional mesh structure, which can effectively reduce the occurrence of cavitation and noise emissions. Ye et al. [96] presented a method of increasing the number of intake valves to increase pump chamber pressure and suppress cavitation. The flow rate of a piezoelectric pump with 6 inlet and 2 outlet valves is four times that of a PP using 4 inlet and 4 outlet valves. Chen et al. [97] studied the effect of wheel valves on air resistance in piezoelectric micro-pumps and found that reducing the thickness or diameter ratio of the wheel valve can reduce the risk of air blockage. When the thickness is 0.02 mm, the diameter ratio is 1.2, and the opening height of the wheel check valve is 252 µm; within a given bubble volume, the probability of air blockage is less than 2%.

### 4.2. Back-Flow Suppression Methods

**Figure 12:**
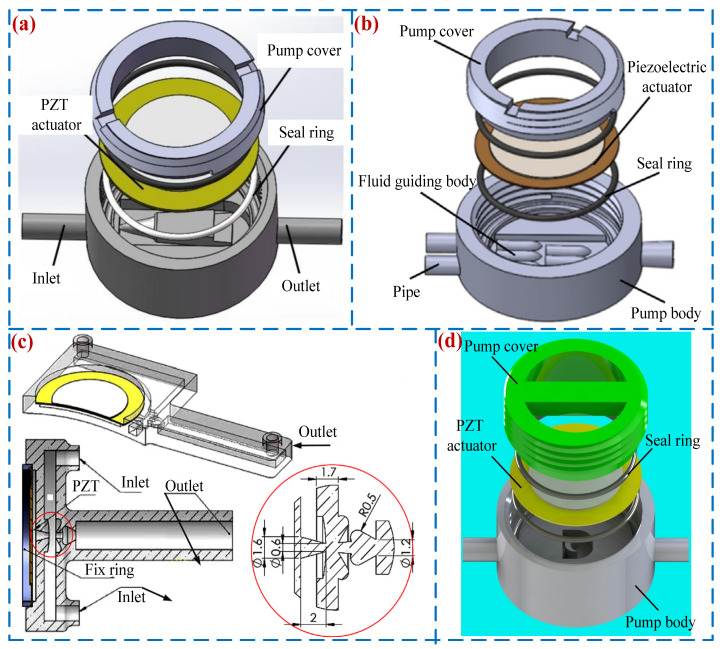
Back-flow suppression methods: (**a**) a VLPP with dome composite structures by He et al. [38], (**b**) a double-outlet VLPP with a fluid guiding body by Zhang et al. [100], (**c**) a new structure micro-pump by Tran et al. [101], and (**d**) a piezoelectric pump with a crescent-shaped structure by Zhao et al. [102].

The back-flow phenomenon has always been a key factor affecting the output performance of PPs, especially in VLPPs. To solve this problem, many scholars have carried out relevant work [98,99]. He et al. [38] presented a VLPP with dome composite structures (see Figure 12a) that can effectively reduce back-flow and improve the output performance of the PP; its peak output pressure and flow rate were 0.67 kPa and 220.6 mL/min, respectively. Zhang et al. [100] designed a double-outlet VLPP with a fluid guiding body (see Figure 12b) that can attenuate liquid back-flow energy and alleviate the back-flow problem of VLPP; its peak flow rate of 167.8 mL/min was achieved when the voltage was 210 V_p-p_ at 49 Hz. Tran et al. [101] proposed a novel VLPP (see Figure 12c) whose flow tube is a Tesla coupled nozzle that can enhance vortices, reduce the liquid back-flow, and improve the output performance of the PP. Zhao et al. [102] presented a VLPP with a crescent-shaped structure (see Figure 12d) that can effectively weaken the back-flow phenomenon and improve the output performance of the PP; its peak flow rate was 286 mL/min.

### 4.3. Optimization of PZT Vibrator

**Figure 13:**
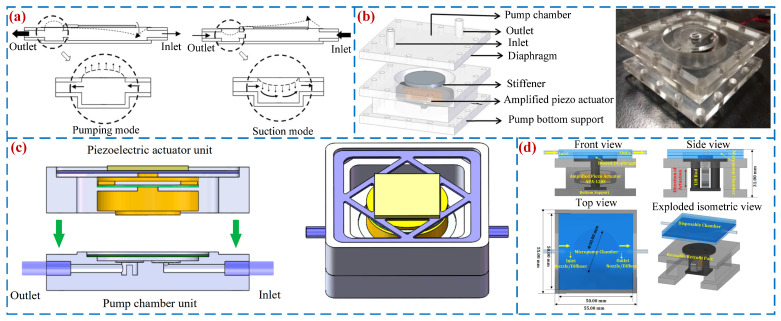
Back-flow suppression methods: (**a**) an innovative one-sided actuating piezoelectric valve-less micro-pump with a secondary chamber by Ma et al. [104], (**b**) a disposable micro-pump with a retrofit piezo stack actuator by Mohith et al. [105], (**c**) a piezoelectric resonance pump by Wang et al. [106], and (**d**) a valve-less micro-pump with a disposable chamber by Mohith et al. [107].

Being the power source of PPs, the structure and installation method of PZT vibrators can affect their amplitude, thereby affecting the flow rates and back pressures of PPs [103]. Ma et al. [104] optimized two series connected PZT vibrators (see Figure 13a), including single-sided driven PZT vibrators and circular assisted PZT vibrators, which are driven asynchronously to improve the speed of liquid delivery, with a peak flow rate of 4.1 mL/s. Mohith et al. [105] presented a disposable medical micro-pump based on a diamond shaped amplification mechanism (see Figure 13b). The driving part of the pump is a diamond-shaped amplification piezoelectric driver; a peak flow rate of 3.4 mL/min was achieved when the voltage was 150 V_p-p_ at 5 Hz. Wang et al. [106] designed a piezoelectric resonance pump based on a flexible support (see Figure 13c). The pump separated the pump chamber diaphragm and the driving unit and used the resonance principle to expand the diaphragm amplitude. Its peak output pressure and flow rate were 85.2 kPa and 213.5 mL/min. Mohith et al. [107] developed a novel biomedical VLPP (see Figure 13d) driven by a bent amplification piezoelectric actuator. The pump was equipped with convex diaphragms in the pump chamber and a piezoelectric actuator, which effectively improved pumping efficiency; its peak flow rate was 7.192 mL/min.

### 4.4. Device Efficiency

Working efficiency is one of the most important performance indicators of PPs, as it directly affects the feasibility and practicality of PPs in applications. Under the same energy consumption, high-efficiency PPs can achieve larger flow rates and higher output pressures. The structure of PPs, the performance of piezoelectric vibrators, and the precise control of driving parameters are the key factors influencing the working efficiency of PPs. In order to enhance the efficiency of PPs, scholars have conducted numerous studies.

Bian et al. [32,33] has reduced the generation of vortices through structural optimization, making the fluid flow more smoothly and significantly improving the pump efficiency. The MCP proposed by Hu et al. [84] can increase the pump flow rate. The PP with a vortex linear valve proposed by Yan et al. [71] can reduce liquid back-flow, and the lagging of valves may even occur, thus enhancing the pump efficiency. The efficient driving of fluid can be achieved through the structural optimization of piezoelectric vibrators and the use of higher-performance piezoelectric materials. The thin layer unimorph ferroelectric drive fabricated by Yoon et al. [108] can achieve a larger axial displacement. In recent years, the emergence of new materials such as Lead Magnesium Niobate-Lead Titanate (PMN-PT) have been able to maintain high piezoelectric performance while having better flexibility. The application of intelligent control algorithms in piezoelectric pumps has significantly enhanced the efficiency of PPs. Zhang et al. [109] presented a PP that uses a three-pole piezoelectric vibrator for driving and flow self-sensing, which can ensure that the PP always operates at the optimal working frequency and improves the pump efficiency.

## 5. Application of PPs

### 5.1. Chip Cooling

**Figure 14:**
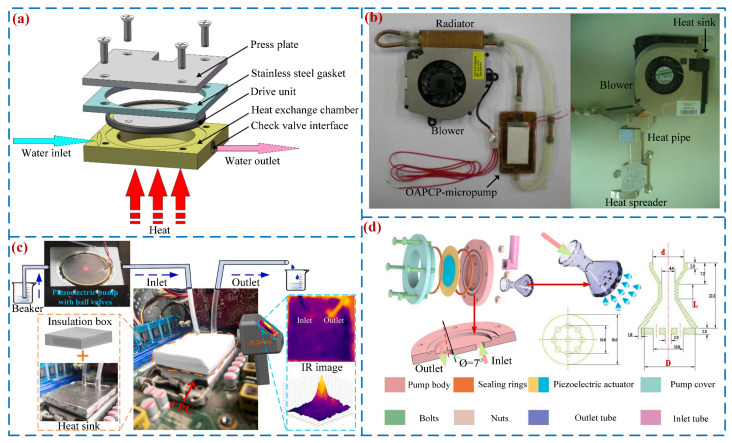
Liquid cooling applications: (**a**) diagram of heat management cooling system by Tang et al. [110], (**b**) circulation cooling system with a one-sided actuating piezoelectric micro-pump by Ma et al. [111], (**c**) a CPU cooling system by Huang et al. [112], and (**d**) a cooling system by Hu et al. [113].

With the continuous development of microelectronics technology and integrated circuit processes, chips are showing a trend towards high integration, leading to a sharp increase in heat flux density. Heat dissipation is a key factor hindering further improvement of chip performance. Liquid cooling systems have been used for chip cooling due to their powerful heat dissipation capabilities. To address the issue of chip heat dissipation, Tang et al. [110] proposed an integrated heat dissipation system with a piezoelectric micro-pump (see Figure 14a), and the wall temperature rise was 47.3 °C when the heating power is 80 W. Ma et al. [111] designed a heat dissipation system that includes a PP and a cold plate radiator (see Figure 14b). Under simulated heat sources of 30 W and 45 W, the total thermal resistance of the system was 0.97 °C/W and 0.967 °C/W, respectively. Huang et al. [112] combined a ball valve PP and a multistage Y-shaped micro-channel to propose a highly integrated heat dissipation system (see Figure 14c) that can control the temperature within 55 °C under extreme loads. Hu et al. [113] presented a PP with the combination of a diffuser/nozzle tube and a Laval tube (see Figure 14d). The results show that this external Laval tube can improve the spray heat dissipation performance of the high-pressure pump. Fan et al. [114] combined jet impingement cooling with PPs to propose a compact liquid cooling system with power consumption of 0.023 W under a heat load of 50 W/cm^2^. Liu et al. [115] proposed a battery thermal management system integrating a piezoelectric pump and a thermoelectric cooler.

### 5.2. Biomedical Applications

**Figure 15:**
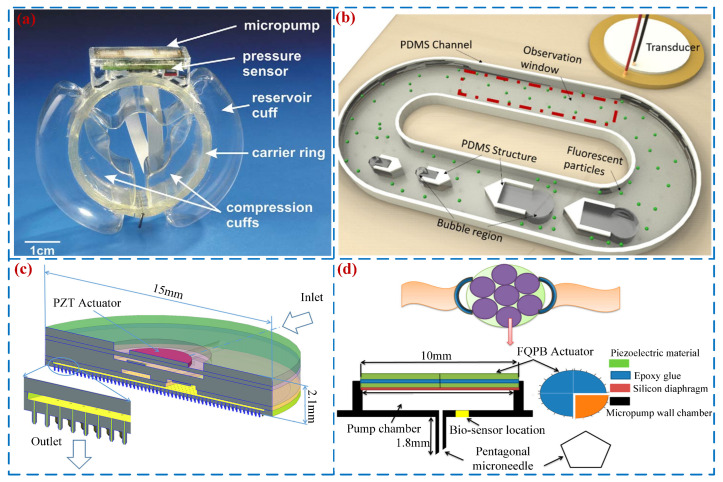
Biomedical applications: (**a**) an artificial sphincter system by Doll et al. [116], (**b**) an acoustic bubble-based bidirectional micro-pump by Gao et al. [117], (**c**) a MEMSs-based drug delivery device by Meshkinfam et al. [118], and (**d**) a blood sampling device by Haldkar et al. [119].

A piezoelectric micro-pump can precisely control flow and achieve low power consumption, minimum return pressure, and bio-safety drive. It was first used to help accurately transport insulin into diabetes patients [80]. Doll et al. [116] proposed a bidirectional micro-pump for a novel artificial sphincter system (see Figure 15a) that can serve as a key component of a new medical prosthesis for treating anal incontinence. Its volume is only 300 mm^3^ with a maximum flow rate of 1.8 mL/min. Gao et al. [117] designed a high-performance bidirectional micro-pump (see Figure 15b). This pump can stably pump biological fluids without damaging cell structure and has a peak flow rate of 1.6 μL/min. Meshkinfam et al. [118] presented a MEMSs (Micro-Electro-Mechanical Systems)-based drug delivery device with an integrated microneedle (see Figure 15c) that is capable of pumping viscous Newtonian liquids. Haldkar et al. [119] proposed a piezoelectric micro-pump for liquid drug delivery (see Figure 15d); a peak flow rate of 1.256 μL/min was achieved when the frequency was 22 kHz. Yan et al. [120,121] invented an ultrasonic atomizer for inhalation therapy using the principle of piezoelectric pumps and tested the performance of the atomizer.

### 5.3. Chemical Applications

**Figure 16:**
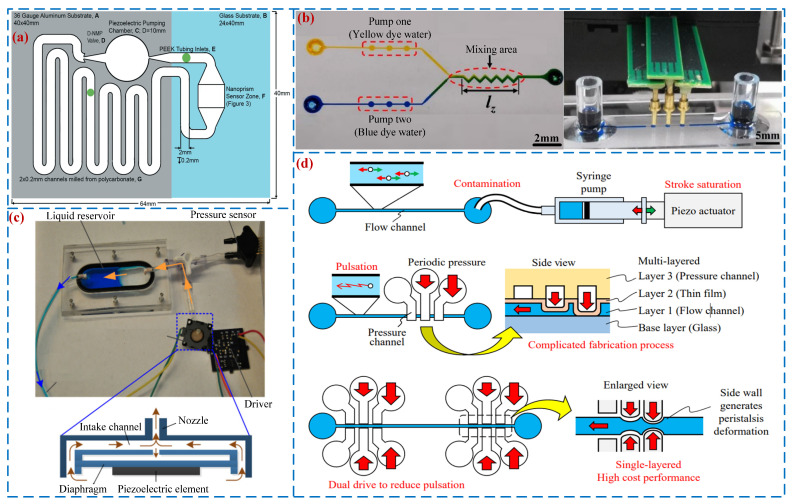
Chemical applications: (**a**) a PP used in rapid real-time recirculating PCR by Haber et al. [122], (**b**) a piezoelectric peristaltic micro-pump by Ma et al. [123], (**c**) a microfluidic pump by Wang et al. [124], and (**d**) a sidewall-driven micro-pump by Atsumi et al. [125].

With the advantages of such a small size and short reaction time, “lab-on-a-chip” has been widely used in chemical analysis. Haber et al. [122] integrated a VLPP with diffuser/nozzle tubes into a microfluidic platform system (see Figure 16a), providing a circulating power source for the chip to perform a real-time polymerase reaction and achieve rapid amplification of the target gene nucleic acid, and the flow rate of 45 μL/min could be obtained under the voltage of 40 V_p-p_. Ma et al. [123] designed a piezoelectric peristaltic micro-pump (see Figure 16b) that can be used for fluid pumping in microfluidic chips. The pump achieved a maximum output pressure of 2.0 kPa and a flow rate of 102 nL/s, and its output resolution was 8.4 nL/s. Wang et al. [124] designed a microblower-based microfluidic pump (see Figure 16c), realizing a peak flow rate of 128 mL/min. Atsumi et al. [125] presented a sidewall-driven micro-pump integrated into microfluidic devices (see Figure 16d) that can achieve precise particle manipulation and is expected to be used for cell manipulation.

### 5.4. Fuel Supply Applications

**Figure 17:**
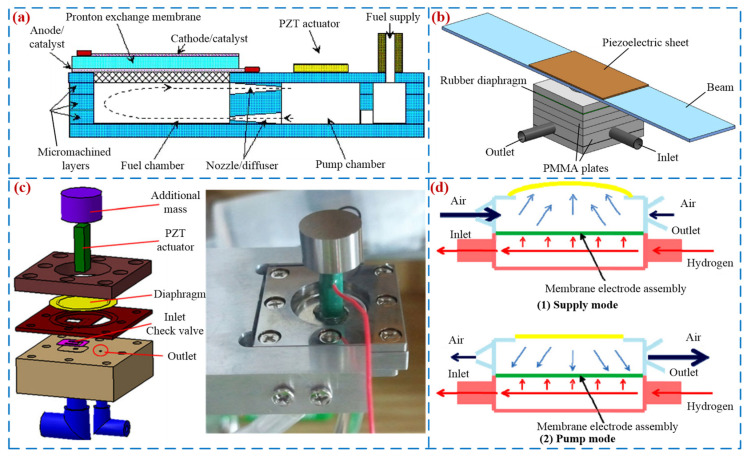
Fuel supply applications: (**a**) a PZT-actuated diaphragm pump by Zhang et al. [127], (**b**) a high flow rate piezoelectric micro-pump by Wang et al. [128], (**c**) a high-output piezoelectric micro-pumps by Park et al. [129], and (**d**) a PZT-PEMFC-ND by Ma et al. [130].

Fuel cells can convert chemical energy into electrical energy, and PPs have low power consumption and stable flow supply, making PPs an effective tool for transporting fuel in fuel cells [126]. Zhang et al. [127] proposed a small fuel cell for piezoelectric pumping of methanol fuel (see Figure 17a), and the performance of the fuel cell was optimal when the pump flow rate was 2.5 mL/min. Wang et al. [128] proposed a piezoelectric micro-pump with high flow rate and high pumping pressure that can be used in fuel delivery systems (see Figure 17b); the maximum flow rate of 105 mL/min and pressure of 23 kPa were obtained at the voltage and frequency of 400 V and 490 Hz. In order to supply liquid fuel in the fuel supply system, Park et al. [129] designed a small volume, high output performance piezoelectric micro-pump (see Figure 17c); the pressure of 14 kPa and the flow rate of 62 mm^3^/s were achieved. Ma et al. [130] invented a proton exchange membrane fuel cell with a piezoelectric actuation structure with a nozzle and a diffuser. The optimal operating temperature and frequency for this PZT-PEMFC-ND pump are 323 K and 180 Hz, respectively, and its power can reach 0.18 Wcm^−2^.

### 5.5. Other Applications

**Figure 18:**
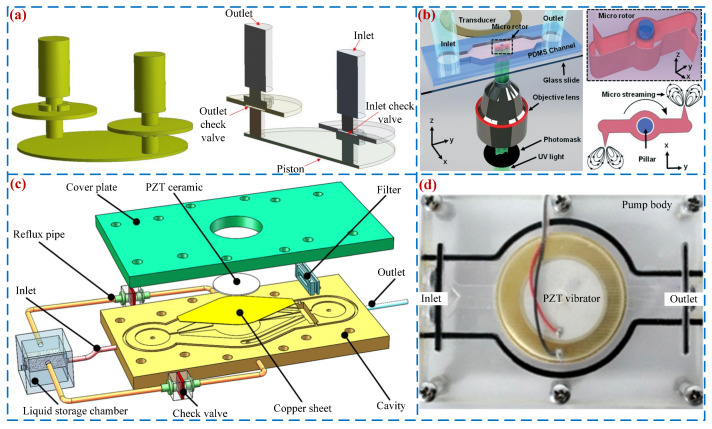
Other applications: (**a**) a small piezoelectric micro-pump by Anh et al. [131], (**b**) a polymeric micro-rotor by Kaynak et al. [132], (**c**) a universal piezoelectric micro-pump by Zhang et al. [133], and (**d**) a VPMP by Sun et al. [134].

Due to their simple structure, low power consumption, and high control accuracy, PPs have found widespread applications in many other fields. Anh et al. [131] proposed a small piezoelectric hydraulic pump that can be used for braking systems of small- and medium-sized unmanned aerial vehicles (see Figure 18a); it obtained a maximum flow rate of 158 mL/min under the frequency of 110 Hz. Kaynak et al. [132] developed a novel micro-acoustic drive motor using a pump cavity driven by a single PZT vibrator (see Figure 18b). The motor can generate vortices at the rotor tip by changing the pump cavity volume, and the local pressure difference causes the rotor to rotate. Its maximum rotor speed of 1200 r/min was obtained under the voltage of 40 V_p-p_. Zhang et al. [133] proposed a precise pumping system capable of self-cleaning based on piezoelectric micro-pumps (see Figure 18c) that can simultaneously achieve stable and long-term directional quantitative filtration and pumping. Sun et al. [134] proposed a novel valve-less piezoelectric hybrid pump (see Figure 18d) that can simultaneously achieve pumping and mixing functions; its maximum flow rate is 12.26 mL/min. Back da Trindade et al. [135] developed a compact flow-batch analyzer equipped with mini piezoelectric pumps and image-based volume control.

**Figure 19:**
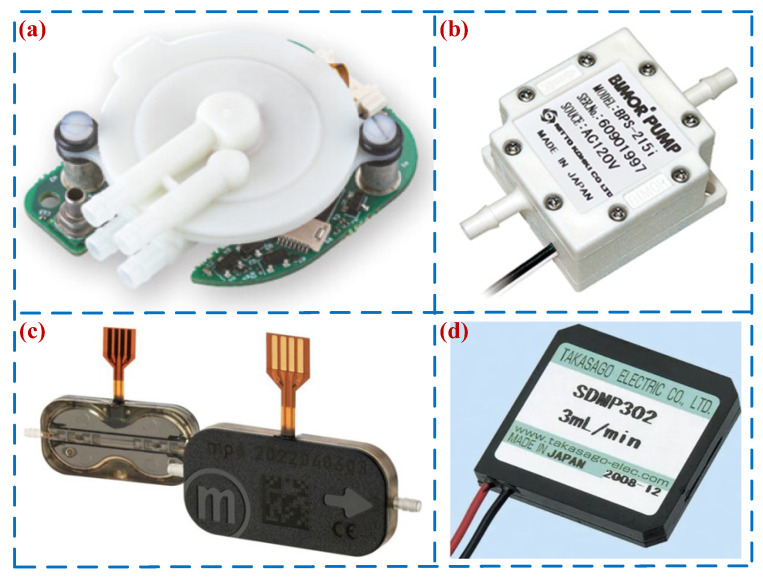
Commercially available PPs: (**a**) TT (LEE) Ventus, (**b**) Bimor, (**c**) Bartels, and (**d**) SDMP302.

In addition, in the field of commercial applications, many companies have successfully launched related PP products, such as TT (LEE) Ventus, Bimor, Bartels, and SDMP302. Figure 19 shows the commercially available piezoelectric pumps. Table 5 lists the parameters of commercially available PPs. As is evident from Figure 19 and Table 5, currently commercially available PPs can achieve high precision and low power consumption and are used in fields such as chemical analysis, drug delivery, fuel control, and biological devices.

## 6. Analysis of the Current Research Status of PPs

**Figure 20:**
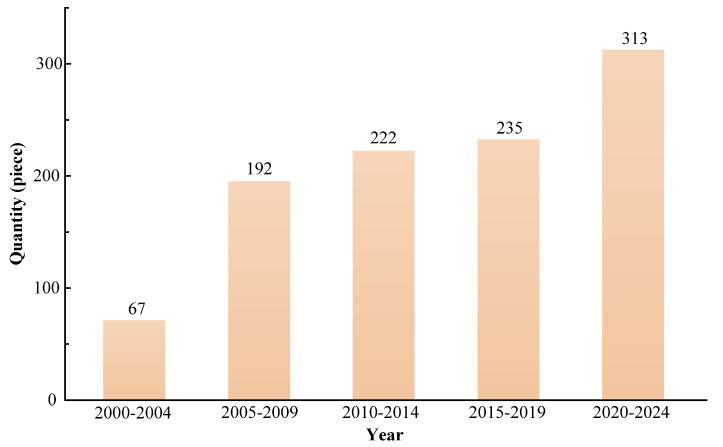
Bar chart of articles on PPs in the past 25 years.

Figure 20 shows the bar chart of articles on PPs in the past 25 years. In this review, we count the number of articles on PPs every 5 years. According to Figure 20, from 2000 to 2024, the total number of articles on PPs was 1029, and the number of articles on PPs showed an increasing trend.

**Figure 21:**
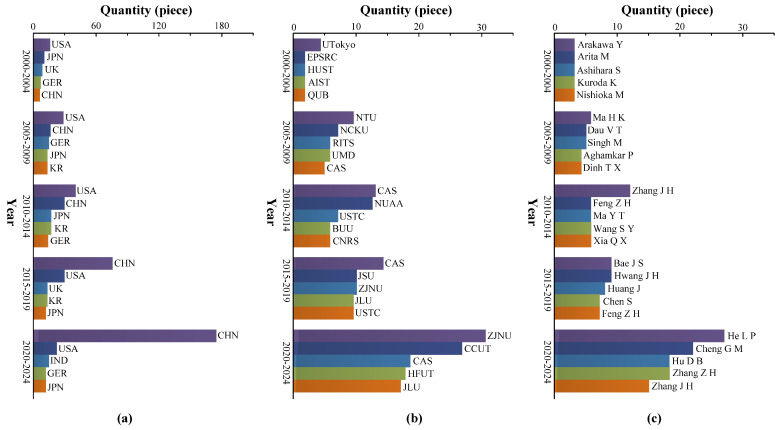
Distribution of research on PPs: (**a**) different countries, (**b**) different institutions, and (**c**) different authors.

Figure 21 shows the distribution of research on PPs. Figure 21a shows the top 5 countries in terms of the number of publications on PPs every 5 years since 2000. As shown in Figure 21a, the United States of America (USA), Japan (JPN), the United Kingdom (UK), and Germany (GER) have been conducting research on PPs for a long time. From 2000 to 2014, USA was the most prominent country in PP research, and from 2015 to 2024, the People’s Republic of China (CHN) had the most research results on PPs. Simultaneously, CHN is the country with the fastest increase in the number of articles in the field of PPs. Figure 21b shows the top 5 institutions in terms of the number of articles on PPs every 5 years since 2000. As shown in Figure 21b, the Chinese Academy of Sciences (CAS) is the institution with the strongest sustainability in research results of PPs, while Zhejiang Normal University (ZJNU) has the fastest increase in research results in the past 10 years. Figure 21c shows the top 5 scholars who have published articles on PPs every 5 years since 2000. Figure 21c shows that different scholars have made outstanding contributions to the research of PPs in each time period.

In summary, with the development of MEMSs and the promotion of PPs, more and more countries, institutions, and scholars have begun to conduct research on PPs, resulting in a significant increase in research achievements related to PPs. The contributions of the Zhang J H team and the Cheng G M team to the field of PPs are important reasons for the rapid increase in research results of CHN’s PPs. The Zhang J H team, including Xia Q X, Huang J, and others, mainly conducted research on VLPPs. The main research results include: a spiral flow tube-type VLPP [136], a VLPP with Y-shaped tubes [31], a VLPP with streamlined flow tubes [32], etc. The Cheng G M team, including He L P, Hu D B, Liu G J, Dong J S, and others, mainly conducted research on VBPPs. The main research results include: a wheeled valve PP [51], a PP with active valve [59], a cantilever valve dual-chamber PP [83], etc. He L P also conducted research on VLPPs [22], mainly focusing on the design and optimization of the internal structure of the pump chamber.

## 7. Summary and Outlook

### 7.1. Summary

This article summarizes the main factors that affect the performance of PPs, including valve structure, flow tube structure, pump chamber quantity, etc. This review summarizes the performance optimization methods of PPs, introduces the application fields of PPs, and analyzes the distribution of research results on PPs. This review can draw the following conclusions:

(1) Based on the “valve” structure, PPs are classifiable as VLPPs, VBPPs, and PPVVSTs. VLPPs have the problem of “back-flow”, resulting in lower work efficiency. The output performance of VBPPs is superior to that of VLPPs, but VBPPs have the problem of “lagging of valve”. PPVVSTs can effectively solve the problems of “back-flow” in VLPPs and the problem of “lagging of valve” in VBPPs, thus further expanding the application of PPs.
(2) Based on the number and arrangement of pump chambers, PPs can be divided into SCPs, multi-chamber series-type PPs, multi-chamber parallel-type PPs, and multi-chamber hybrid-type PPs. Compared with SCPs, MCPs have a significantly improved output performance. PPs can obtain the maximum flow rate when the pump chambers are connected in a series, and the maximum output pressure can be obtained when the pump chambers are connected in a mix.
(3) In order to improve the output performance of PPs, optimization methods proposed by scholars have been summarized for the problems of cavitation and back-flow in PPs. Meanwhile, the structure and installation method of the PZT vibrators have been optimized to improve driving efficiency.
(4) At present, the demand for PPs in the microfluidic field is increasing, especially in areas such as chip cooling, biomedical, chemical analysis, and fuel supply. More and more countries, institutions, and scholars are conducting research in PPs, and CHN is the country with the fastest increase in research results in PPs.

### 7.2. Outlook

Numerous scholars have conducted research on PPs with many achievements, but the following points need to be improved.

(1) Integrated pump body structure

The sealing issue has always affected the accuracy of PPs. Three-dimensional printing technology can integrate the pump body, flow channel, and pump cover in order to avoid liquid leakage. Three-dimensional printing technology will be the mainstream method for producing PPs in the future.

(2) Pumping of high-viscosity medium

PPs are commonly used for pumping liquids, gases, and low-viscosity solutions, while high-viscosity liquid pumping is involved in fields such as solution mixing, fuel supply, and biomedical applications. High-viscosity liquid pumping will be a key direction in the field of PPs, helping to expand the application areas of PPs.

(3) Miniaturization of pump body size

The size of PPs is an important factor affecting their resolution. Currently, PPs are unable to pump fluids with nL level resolution. In the future, micro-scale PPs can be manufactured to pump fluids with nL level resolution.

(4) Diversified application fields

At present, the application of PPs is mainly focused on chip cooling, biomedical, chemical analysis, fuel supply, etc. In the future, PPs are expected to be used for driving deep-sea robots, navigation guidance, and other fields.

## Figures and Tables

**Table 1:** Summary of properties for VLPPs.

First Author and Year	Type of Valve	Structure Material	*V*_p_(L × H × W) (mm^3^)	*U*_p_ (V_p-p_)	*F*_p_ (Hz)	*P*_max_ (kPa)	*Q*_max_ (mL/min)
2013 [28] Tseng	Diffuser/nozzle	Polymethyl Methacrylate (PMMA)	N/A	160	400	5.3	1.2
2017 [29] Aggarwal	Diffuser/nozzle	Si-wafer	10 × 10 × 1	80	1050	3.1	0.355
2013 [31] Huang	Multistage Y-shaped	PMMA	N/A	100	9.0/10.3	0.55	35.6
2020 [32] Bian	Streamlined	Stereolithography Apparatus (SLA)	N/A	100	80	N/A	142.0
2018 [35] Munas	Cross-shaped	PMMA	100 × 60 × 5.5	N/A	100	24	31.15
2019 [38] He	Unsymmetrical chamber	Polylactic Acid (PLA)	70 × 42 × 27	190	45/130	0.67	220.6
2010 [44] Huang	Bionic	PMMA	80 × 35 × 25	100	460	N/A	6.4

**Table 2:** Summary of properties for VBPPs.

First Author and Year	Type of Valve	Structure Material	*V*_p_(L × H × W) (mm^3^)	*U*_p_ (V_p-p_)	*F*_p_ (Hz)	*P*_max_ (kPa)	*Q*_max_ (mL/min)
2020 [47] Woo	Cantilever valve	Poly Carbonate	50 × 50 × 72.6	N/A	110	36.4	91.5
2018 [48] Ye	Bridge valve	PMMA	60 × 60 × 12	400	750	6	187.2
2021 [49] Pan	Ball valve	PMMA	20 × 20 × 22	448	790	15.3	99.6
2016 [50] Zhang	Umbrella valve	N/A	N/A	150	300	3.65	165
2022 [51] He	Wheeled valve	PLA	42 × 42 × 30	220	70	N/A	431.6
2022 [52] Yang	Cantilever valve	PMMA	11 × 11 × 1.5	180	150/50	52	4.5
2016 [56] Zhang	Umbrella valve	PMMA	40 × 40 × 17	300	12/21	104.7	28.71
2015 [57] Ma	Wheeled valve	PMMA	50 × 50 × 12	70	25	N/A	196
2004 [58] Lee	Active	N/A	N/A	N/A	1000	N/A	204
2010 [59] Cheng	Active	PMMA	82.6 × 77.4 × 19	75	70	N/A	140

**Table 3:** Summary of properties for PPVVST.

First Author and Year	Type of Valve	Structure Material	*V*_p_(L × H × W) (mm^3^)	*U*_p_ (V_p-p_)	*F*_p_ (Hz)	*P*_max_ (kPa)	*Q*_max_ (mL/min)
2019 [68] Bao	Compliant	Polydimethylsiloxane (PDMS)	40 × 20 × 4.5	210	80	N/A	3.6
2021 [69] Huang	Flexible	SLA	N/A	100	25	6.16	119.61
2022 [70] Zhou	Cardiac valve-like structure	SLA	N/A	180	12	N/A	44.1
2024 [71] Yan	Vortex linear valve structure	SLA	70 × 50 × 11	180	12	N/A	44.3

**Table 4:** Summary of properties for SCPs and MCPs.

First Author and Year	Type of Pump	Structure Material	*V*_p_(L × H × W) (mm^3^)	*U*_p_ (V_p-p_)	*F*_p_ (Hz)	*P*_max_ (kPa)	*Q*_max_ (mL/min)
2021 [77] Huang	SCP	Random Polypropylene Copolymer (PPR)	28 × 41 × 41	240	22/23	0.17	27.5
2018 [35] Munas	SCP	PMMA	100 × 60 × 5.5	N/A	100	24	31.15
2011 [79] Choi	SCP	Aluminum	50 × 50 × 30	320	45	0.93	23.0
2020 [80] Dereshgi	SCP	PMMA	N/A	45	20	N/A	35.4
2023 [81] Wang	SCP	PPR	N/A	100	47	0.843	235.56
1995 [82] Olsson	MCP	N/A	N/A	80	540	16.5	16.0
2005 [83] Kan	MCP	PMMA	N/A	50	200	9.0	3.0
2024 [84] Hu	MCP	UV curable resin	85 × 50 × 20	210	50	19.38	466.0
2022 [85] Liu	MCHP	PMMA	53.8 × 22 × 5	170	550/450	140.0	110.0
2015 [74] Zhang	MCPP	PMMA	N/A	150	80/400	16.1	469.2
2020 [86] Lu	MCHP	PMMA	270 × 55 × 28	60	105	60.2	251.1

**Table 5:** The parameters of commercially available piezoelectric pumps.

Brand	U_p_ (V_p-p_)	F_p_ (Hz)	Q_max_ (mL/min)	P_max_ (kPa)	Power Consumption (W)	Origin	Application Fields
TT Ventus	48	1–200	50	100	N/A	United Kingdom (UK)	Chemical analysis
Bimor	120	60	30	15	1	Germany (GER)	Drug delivery, Fuel control
Bartels	2.7–5.5	N/A	6	55	0.2	GER	Biomedical devices
SDMP302	60–250	60	3	40	0.029	Japan (JPN)	All-purpose
